# Epidemiology, genetic diversity, and association of canine circovirus infection in dogs with respiratory disease

**DOI:** 10.1038/s41598-022-19815-z

**Published:** 2022-09-14

**Authors:** Wichan Dankaona, Emmita Mongkholdej, Chakkarin Satthathum, Chutchai Piewbang, Somporn Techangamsuwan

**Affiliations:** 1 Department of Pathology, Faculty of Veterinary Science, Chulalongkorn University, Bangkok, 10330 Thailand; 2 Animal Virome and Diagnostic Development Research Group, Faculty of Veterinary Science, Chulalongkorn University, Bangkok, 10330 Thailand; 3 Division of Dentistry and Maxillofacial Surgery, Kasetsart University Veterinary Teaching Hospital, Bangkok, 10900 Thailand

**Keywords:** Molecular biology, Infection, Respiratory tract diseases, Viral infection

## Abstract

Although canine circovirus (CanineCV)-associated with gastroenteritis has been well documented, the virus is also detectable in the respiratory discharge of dogs with respiratory disease. In this study, an epidemiological approach was used to explore the association between the presence of CanineCV and respiratory symptoms in dogs. Respiratory swabs were collected from 76 healthy dogs and 114 dogs with respiratory illness and tested for CanineCV using conventional PCR (cPCR). Furthermore, lung tissues collected from 15 necropsied dogs showing pneumonia were tested using the real-time PCR (qPCR) and in situ hybridization (ISH) technique. A total of 8.95% (17/190) of dogs were CanineCV positive, with a significant association (*p* = 0.013) in dogs with respiratory signs. Four necropsied dogs were qPCR positive with the CanineCV-DNA labeling localized in tracheobronchial lymphoid cells (3/4), pulmonary parenchyma, capillary endothelia, and mononuclear cells harboring in alveoli (2/4). Full-length genome sequences of seven CanineCV strains were analyzed, indicating that the detected CanineCV genome clustered in the CanineCV-4 genotype. Genetic recombination was also evident in the replicase (*Rep*) gene. Although the role of CanineCV primarily affecting lung lesions could not be determined from this study, the presence of CanineCV DNA in pulmonary-associated cells indicated the potential association of the virus with canine respiratory disease; thus, linking causality must be examined in further studies.

## Introduction

Canine circovirus (CanineCV) is a small, non-enveloped, closed-circular, single-stranded DNA (ssDNA) virus belonging to the genus *Circovirus*, family *Circoviridae.* The CanineCV genome consists of ~ 2 kb of nucleotide length and contains two major putative open reading frames (ORFs), placed in the opposite orientation^1^. ORF1 encodes a replication-associated protein (Rep), whereas ORF2 encodes a viral capsid protein (Cap). A previous study indicated that the Cap protein was associated with immune responses, as demonstrated by the seroconversion of specific antibodies to the recombinant CanineCV Cap following experimental virus inoculation^2^.

*Circovirus* has been identified in various avian and mammalian species, including canary circovirus (CaCV)^3^, goose circovirus (GoCV)^4^, gull circovirus (GuCV)^5^, beak and feather disease virus (BFDV)^6^, duck circovirus (DuCV)^7^, pigeon circovirus infection (PiCV)^8^, porcine circovirus (PCV)^9,10^, whale circovirus (BWCV)^11^, mink circovirus (MiCV)^12^, bear circovirus (BearCV)^13^, and canine circovirus (CanineCV)^1^. In the porcine respiratory disease complex (PRDC), PCV type 2 (PCV2) is considered one pathogen that can cause respiratory diseases in pigs. This virus can be detected in oropharyngeal swabs (OSs), nasal swabs (NSs), blood and feces of infected pigs^14,15^. Moreover, a previous study found that inoculated pigs with nasal and oral secretions deriving from PCV2-infected pigs had viremia, seroconversion, and histopathological lesions of PCV2 infection^16^. These findings suggest that respiratory secretions could be the potential source of *Circovirus* transmission.

CanineCV was first discovered in dogs in the US in 2012^1^. The virus was found in serum samples from six out of 205 dogs (2.9%) with an unknown history. Subsequently, CanineCV was found in dogs exhibiting systemic vasculitis and hemorrhage^17^. Since then, epidemiological and pathological studies have investigated the association between CanineCV and related diseases, and it has been proposed that CanineCV might be associated with hemorrhagic gastroenteritis and diarrhea^2,18–22^. CanineCV coinfection with other enteric pathogens, especially canine parvovirus-2, has been frequently reported, suggesting a synergistic role of CanineCV in disease development^23–25^. Besides the gastrointestinal system, the CanineCV genome has been detected in the oronasal secretions of dogs with respiratory diseases infected both naturally and experimentally^2,26,27^, implying that canine respiratory tissue could be a tissue site of viral persistence.

To date, the occurrence and association between CanineCV and gastroenteritis in dogs have been widely described, whereas information regarding CanineCV in respiratory diseases in dogs remains limited. Therefore, this study aimed to determine the occurrence and association of the presence of CanineCV genome in dogs with respiratory diseases in Thailand through genome-based and histopathological investigations. The tropism of the CanineCV was presented in lung tissue using the in situ hybridization (ISH) technique. Additionally, complete genomes of CanineCV strains obtained from this study were characterized and subsequently analyzed using phylogenetic and recombination analyses.

## Results

### Demographic data of swab-sampled dogs

Of the 190 dogs, 76 were clinically healthy (40%), and 114 suffered from respiratory diseases (60%). Classified by age, 57 dogs (30%) each were juniors and adults, and 76 (40%) were seniors. The average age was 5.2 years (range 2 months–16 years), with 72 (37.9%) male dogs and 114 (60%) female dogs. The most common dog breed was mixed breed (40.5%, 77/190). For sterilization status, there were 79 (41.6%) intact and 100 (52.6%) neutered dogs. Regarding clinical information, the most common respiratory symptoms were sneezing and/or nasal discharge. The breeds of studied dogs varied and are shown in Supplementary Table S1.

### Detection of CanineCV and other respiratory viruses in clinical samples

The overall occurrence of PCR-positive CanineCV was 8.95% (17/190), consisting of 2.63% (2/76) from the healthy group and 13.16% (15/114) from the respiratory illness group (Table 1 and Supplementary Table S1). Positive rates of CanineCV detection from each sampling site showed 23.53% (4/17) for NSs, 17.65% (3/17) for OSs, and 58.82% (10/17) for both NSs and OSs. However, there was no statistically significant difference in CanineCV detection between sampling sites (*p* = 0.541). Among CanineCV-positive dogs, nine dogs (52.94%) were co-detected with CaHV-1 (*n* = 2), CDV (*n* = 2), CRCoV (*n* = 2), CAdV-2 (*n* = 1), and triple-detected with CaHV-1 and CRCoV (*n* = 1), whereas eight dogs (47.06%) were infected only with CanineCV and two of them were derived from the healthy group. Most of the CanineCV-positive dogs were junior (*n* = 10), female (*n* = 12), mixed-breed (*n* = 8), and intact (*n* = 10).

**Table 1:** Molecular detection of Canine circovirus (CanineCV)-positive dogs with and without other respiratory viruses from swab samples.

Virus detection^†^	Cases
CanineCV	8^‡^ (47.06%)
**Co-detection**	9 (52.94%)
CanineCV + CaHV-1	2
CanineCV + CDV	2
CanineCV + CRCoV	2
CanineCV + CPIV	1
CanineCV + CAdV-2	1
CanineCV + CaHV-1 + CRCoV	1
Total	17

^†^Canine herpesvirus 1 (CaHV-1), canine distemper virus (CDV), canine respiratory coronavirus (CRCoV), canine parainfluenza virus (CPIV), and canine adenovirus type 2 (CAdV-2).

^‡^The two positive CanineCV dogs in healthy group (H026, H070) were included, while other 15 positive CanineCV dogs were respiratory-suffering dogs.

Apart from CanineCV detection, common canine infectious respiratory viruses were identified in the dogs suffering from respiratory illness (*n* = 56). Single viral detection was evident in 45 dogs, with the highest occurrence being CaHV-1 (*n* = 17). Double viral detection was found in 10 dogs, with the highest occurrence being CaHV-1 and CDV (*n* = 6). Triple viral detection was found in only one dog, represented as PCR-positive for CDV, CRCoV, and CAdV-2 (Supplementary Table S2).

### Association of age and respiratory illness with detected CanineCV

CanineCV was detected in 17.54% (10/57) of junior dogs and to a lesser extent in adult (10.53%, 6/57) and senior dogs (1.32%, 1/76). There was a significant association between the dogs’ classified age range and CanineCV detection (*p* = 0.005), but the significant association was found only in junior dogs (*p* = 0.007). The odds ratio between junior (age < 1.5 years) and older (age ≥ 1.5 years) dogs was 3.83 (95% CI 1.378–10.645) (Table 2). However, associations between sex, breed, sterilization status, sampling route, and positive CanineCV were not evident (Supplementary Table S3). A significant association between respiratory symptoms and CanineCV detection was observed (*p* = 0.013), revealing an odds ratio of 5.606 (95% CI 1.244–25.272) (Table 2). However, other respiratory viruses were co-detected with CanineCV-positive dogs (52.94%; 9/17) (Table 1 and Supplementary Table S1). To determine whether the presence of these viruses in CanineCV-positive dogs had an effect on their respiratory symptoms, Fisher’s exact test was performed and revealed that there was no association between the presence of these viruses and respiratory signs in CanineCV-positive dogs (*p* = 1.00) (Supplementary Table S3).

**Table 2:** The odds for age of dogs and for healthy and respiratory groups.

CanineCV	Age		Disease status		Total
	< 1.5 years	≥ 1.5 years	Healthy	Respiratory	
Positive	10 (17.54%)	7 (5.26%)	2 (2.63%)	15 (13.16%)	17
Negative	47 (82.46%)	126 (94.74%)	74 (97.37%)	99 (86.84%)	173
Odds	0.2128	0.0556	0.027	0.1515	
Total	57	133	76	114	190

### Genomic sequence analysis

Seven selected full-length genomes were sequenced and analyzed. Each CanineCV genome obtained in this study consisted of 2,063 nucleotides, which contained two major ORFs. ORF1 was the *Rep* gene (nt 1–912), which encoded 303 amino acids of the Rep protein, and OFR2 was the *Cap* gene (nt 1116–1928), which encoded 270 amino acids for the Cap protein. The thermodynamic stem-loop sequence (TAGTATTAC) was located at nt 2012–2020.

The nucleotide sequence identity shared among CanineCV Thai strains was 95.2–100% for the full-length genome, 95.0–100% for the *Rep* gene, and 93.6–100% for the *Cap* gene. The amino acid identity of the Rep and Cap proteins among CanineCV Thai strains was 95.7–100% and 94–100%, respectively (Supplementary Table S4).

### Phylogenetic and recombination analyses

**Figure 1:**
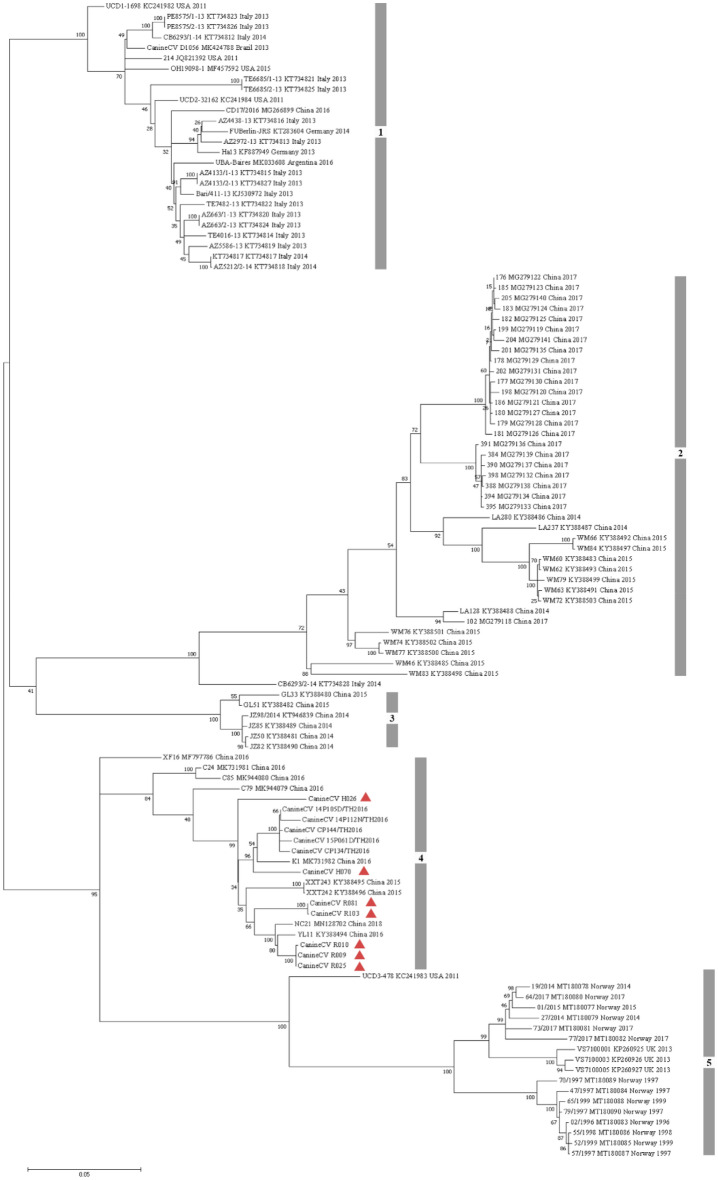
Maximum likelihood phylogenetic tree reconstructed using the full-length canine circovirus (CanineCV) genome. The reliability of the phylogeny was evaluated using 1,000 bootstrap test replications (red triangle represents the CanineCV strains from this study).

Seven full-length genome sequences obtained from this study (MZ826142- MZ826148) were compared with the sequences available in the GenBank database (Fig. 1) by constructing a phylogenetic tree. Overall, CanineCV was divided into five genotypes. The CanineCV-1 genotype was composed of various CanineCV strains detected in the US, Italy, Brazil, Germany, Colombia, and Argentina, as well as some strains found in China. The CanineCV-2 and CanineCV-3 genotypes included most of the strains found in China. The CanineCV strains obtained in the present study were grouped into the CanineCV-4 genotype, together with the previously discovered CanineCV Thai strains in 2016 and some strains found in China. We found that our CanineCV_R009, R010, and R025 strains were genetically identical; they were grouped together and created monophyletic clade closed to the CanineCV strain YL11 (KY388494) found in China. The CanineCV_R081 and R103 strains were clustered with the CanineCV strain NC21 (MN128702) from China. CanineCV_H070 was separated to another neighbor clade, which contained previously described CanineCV Thai strains and the strains found in China. Lastly, the CanineCV_H026 strain had been genetically dispersed from other Thai strains that served as predominant phylogenetic clade. CanineCV strains detected in foxes in the UK and Norway were grouped into the CanineCV-5 genotype.

**Figure 2:**
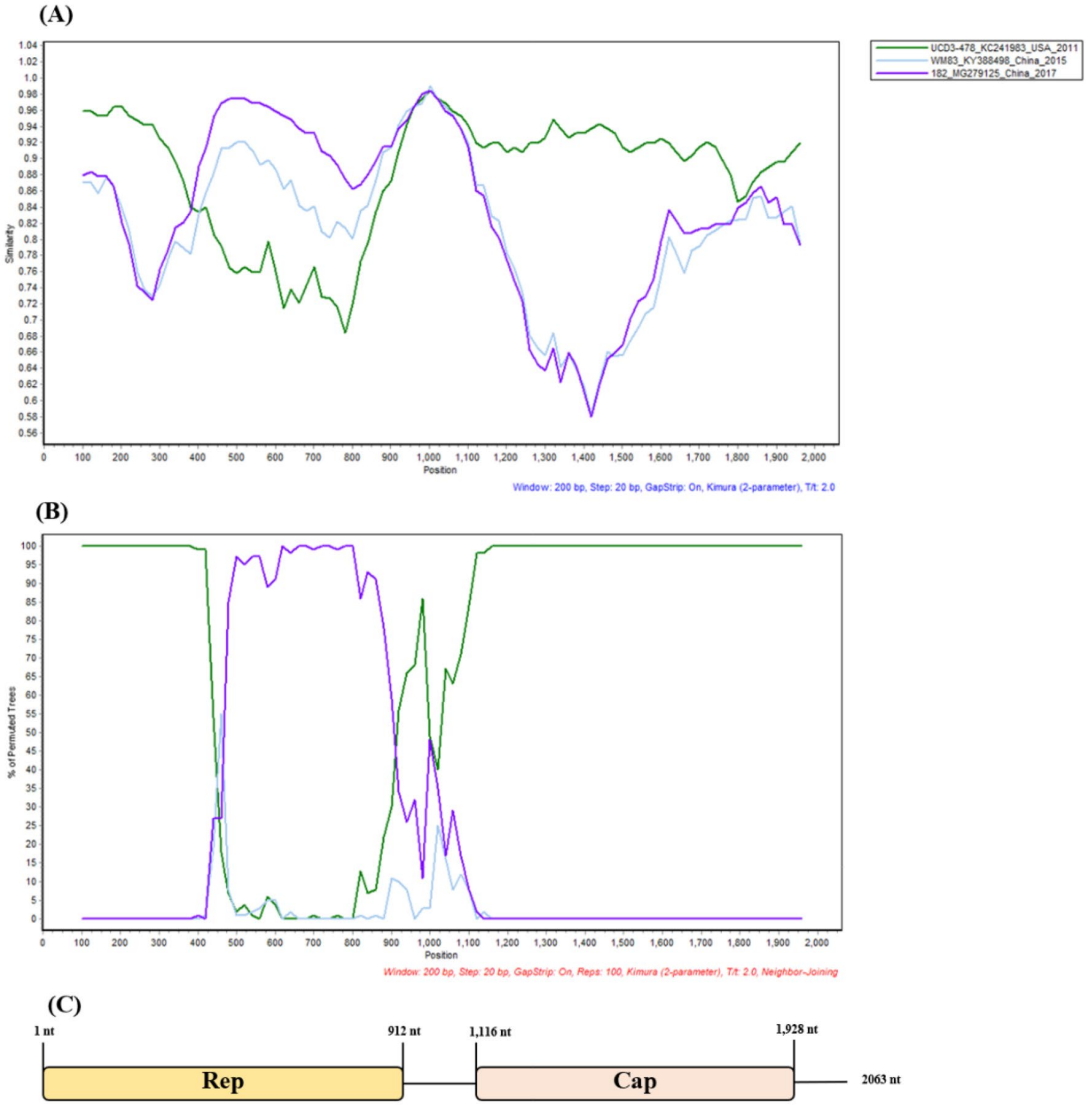
Recombination analysis. (**A**) Nucleotide similarity plot and (**B**) BootScan analysis showing potential breakpoints for canine circovirus (CanineCV) H070 (nt 354–1071). CanineCV H070 was used as a query sequence. Representative parent sequences were CanineCV UCD3-478 (green) and CanineCV 182 (purple). CanineCV WM83 (blue) was used as an outgroup. The Y-axis represents the proportion of nucleotide identity in the SIMPLOT analysis (**A**), and the Y-axis represents the percentage of permutated trees in the BootScan analysis (**B**). The X-axis for both analyses refers to the nucleotide positions along the full-length genome sequence. Analysis was performed with a window size of 200 bp and a step size of 20 bp **(C)**. The CanineCV genomic structure was diagrammed.

To explore the genetic recombination of our CanineCV strains in the present study, recombination analysis using the RDP was conducted based on the complete genome of the detected CanineCV and previously described CanineCV strains. Ten potential recombination strains were detected; whereas only one sequence found in this study, the CanineCV_H070 strain, was found to be a potential recombinant. The CanineCV H070 strain had a potential recombination breakpoint, which was confirmed by positive results with a *p*-value ≤ 10^–10^ in six out of seven algorithms (Supplementary Table S5). The CanineCV strain UCD3-478 (KC241983), which was detected from a dog in the US, and the CanineCV strain 182 (MG279125), originating from a dog in China, served as a major and minor parent of CanineCV_H070, respectively. Analysis by SIMPLOT software confirmed the recombination event in the CanineCV_H070 strain and revealed that it had high nucleotide similarity to the CanineCV strain UCD3-478 (green) in the early region of the *Rep* gene and the entire *Cap* gene, whereas the middle region toward the end of the *Rep* gene had nucleotide similarity to the CanineCV strain 182 (purple) (Fig. 2A). BootScan analysis confirmed the recombination breakpoint to be located between nt 354–1071 in the *Rep* gene (Fig. 2B). The nucleotide sequence amplified from primer pairs covering the recombination breakpoint and phylogenetic trees based on the recombination sites support the recombination events (Supplementary Figs. S1 and S2).

### Screening of CanineCV in tissues by PCRs

Fifteen lung samples from dogs that died from bronchopneumonia were screened for the presence of CanineCV DNA. We found that four dogs (26.67%) were positive for CanineCV on cPCR and negative for other CIRDC viruses (Table 3). The tissue viral loads of lungs and other respiratory-related tissues (trachea, bronchus, tracheobronchial lymph node) were then estimated by qPCR; the results indicated that the CanineCV viral loads were highest in the lungs (dog nos. 1 and 3) and localized lymph nodes (dog nos. 2 and 4) and varied among infected cases. Lung tissues of all positive cases revealed qPCR Ct values ranging from 24.02 to 25.36. Varied amounts of CanineCV loads in other organs are described in Table 3 and Supplementary Table S6.

**Table 3:** Detection of canine circovirus (CanineCV) by using conventional PCR (cPCR), real-time PCR (qPCR), and in situ hybridization (ISH) in various respiratory tissues of CanineCV-positive necropsied dogs.

Animals	Samples	Methods		
		cPCR	qPCR (Ct value)	ISH
Dog No. 1	Trachea	+	N/A	−
	Bronchus	+	N/A	−
	Lung	+	+ (25.36)	+
	Tracheobronchial lymph node	+	+ (27.51)	+
Dog No. 2	Trachea	−	+ (22.37)	−
	Bronchus	−	−	−
	Lung	+	+ (24.02)	−
	Tracheobronchial lymph node	+	+ (15.94)	−
Dog No. 3	Trachea	−	−	−
	Bronchus	−	−	−
	Lung	+	+ (24.18)	−
	Tracheobronchial lymph node	+	+ (25.75)	+
Dog No. 4	Trachea	+	+ (22.51)	−
	Bronchus	+	+ (23.46)	−
	Lung	+	+ (24.24)	+
	Tracheobronchial lymph node	+	+ (22.19)	+

+ positive, − negative, *N/A* no data available.

Because CanineCV could be detected in dogs without respiratory problems, we further investigated the presence of the virus in a non-respiratory disease control group composed of dogs that had died from cardiovascular failure (n = 13) and accidental traumatic injury (n = 15). It was found that none of these cases was positive for CanineCV by cPCR.

### Pathological examination and CanineCV localization in lung sections

**Figure 3:**
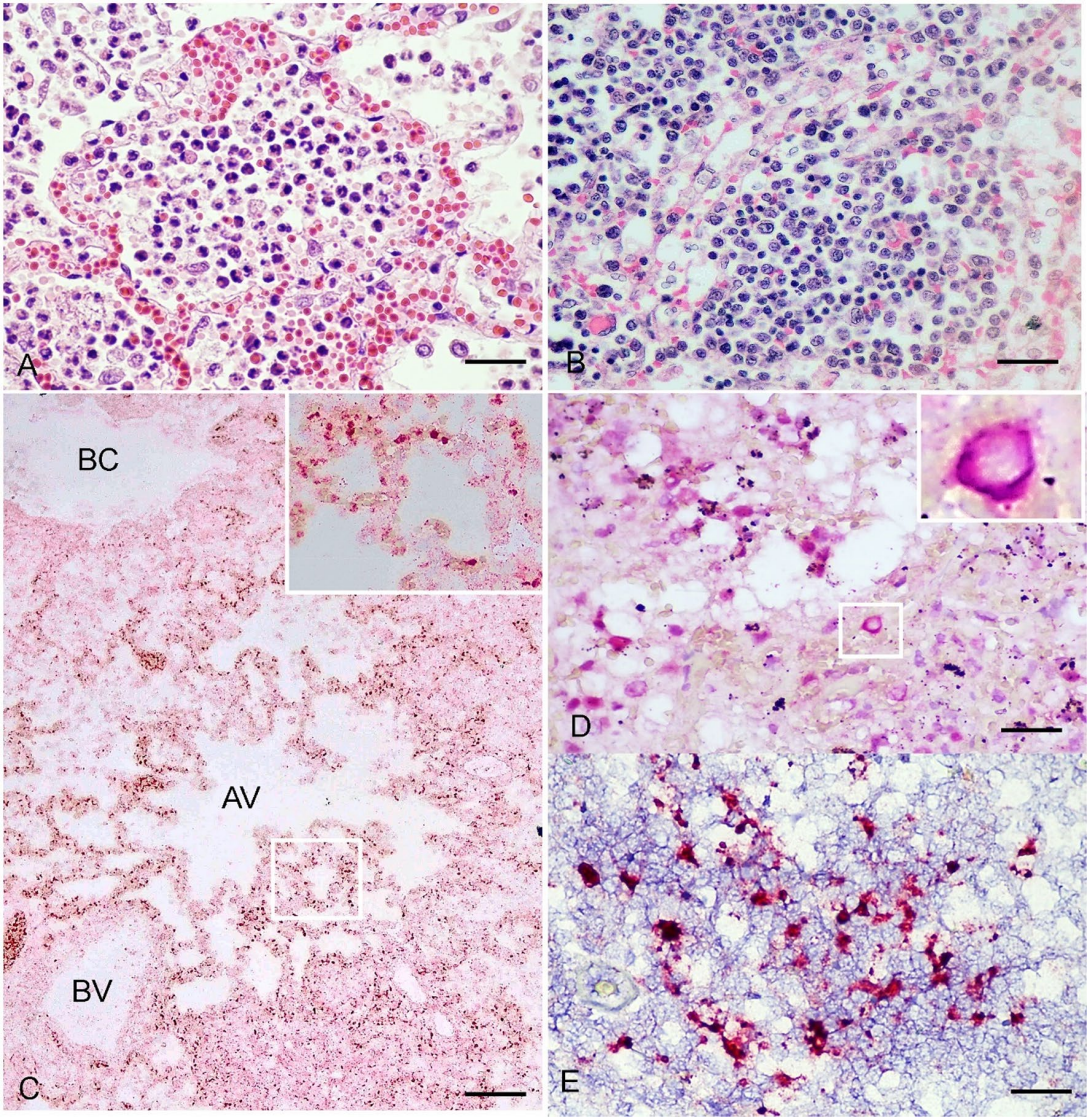
Canine circovirus (CanineCV) infection. Leakage of erythrocytes, degenerated neutrophils, and pulmonary alveolar macrophages were presented in the interstitium and alveoli (**A**). Histiocytic lymphadenitis of tracheobronchial lymph node (**B**). CanineCV DNA labeling (dark-red precipitates) were diffusely presented within pulmonary parenchyma (**C**). Nuclear signals were intensely labeled within cells resembling to pneumocytes and the endothelium of capillary vessels (inset). BC = bronchiole; AV = alveolar space; BV = pulmonary blood vessel. Strong positive cytoplasmic signals of CanineCV nucleic acid were shown in the cytoplasm of mononuclear cells that infiltrated into the lung parenchyma (**C**, inset). Labeling of CanineCV DNA was localized within mononuclear cells residing in tracheobronchial lymph nodes (**D**, **E**). Bar: 50 µm (**A**,**B**,**D**,**E**) and 150 µm (**C**).

To illustrate the localization of CanineCV in the respiratory system, CanineCV-positive lung samples subsequently underwent ISH. Histologically, the CanineCV-positive dogs exhibited a variety of severities of generalized hemorrhagic pyogranulomatous pneumonia (2/4), multifocal hemorrhagic pneumonia with histiocytic infiltration (1/4), and severe diffuse suppurative, hemorrhagic, and necrotic bronchiolitis and alveolitis (1/4) (Fig. 3A). Multiple bacterial clumps were also found associated with degenerated neutrophils and pulmonary alveolar macrophages (3/4). Various inflammation severities with hemorrhagic histiocytic lymphadenitis of tracheobronchial nodes were also indicated (4/4) (Fig. 3B) (Supplementary Table S6). Among the CanineCV PCR-positive dogs, lung samples of two dogs revealed positive CanineCV DNA labeling (dog nos. 1 and 4; Table 3). The positively labeled cells were diffuse within pulmonary parenchyma and in alveolar spaces, multifocally. Specifically, the positive CanineCV nucleic acid labeling was intensely presented within the nucleus of alveolar lining cells and endothelial cells of capillary blood vessels (Fig. 3C). The DNA signals were also labeled in the cytoplasm of infiltrated mononuclear cells resembling pulmonary alveolar macrophages or lymphocytes. The hybridization signals were mostly observed in the pulmonary areas where they presented necrotic alveolitis (Fig. 3D). The CanineCV DNA labeling was intensely localized within the cytoplasm of lymphoid cells in lymphoid follicles of the tracheobronchial lymph node (3/4) (Fig. 3E). Neither tracheal nor bronchial apparatus revealed a positive hybridization signal, nor did the in negative controls (Supplementary Figure S3).

For 11 CanineCV-negative lung samples, bacterial-associated pneumonia was observed, including severe fibrinosuppurative bronchopneumonia (3/11), severe diffuse hemorrhagic suppurative bronchopneumonia (3/11), and multifocal extensive fibrinohemorrhagic necrotizing pneumonia (2/11). Among these dogs, pyogranulomatous lymphadenitis of tracheobronchial lymph nodes was identified (3/11). The lung lesions of the remaining dogs revealed mild fibrinous pneumonia with histiocytic lymphadenitis (3/11). However, bacterial identification was not performed to confirm the etiology of pneumonia.

## Discussion

*Circovirus* is a viral pathogen that can cause various diseases in infected hosts, such as gastrointestinal-, neurological-, immunological-, and respiratory-related diseases^9,20,22,28,29^. Among them, *Circovirus*-associated respiratory disease has been reported in several infected hosts^30,31^, indicating the possible role of this virus in respiratory disease. Regarding CanineCV, the virus has also been frequently detected in dogs exhibiting respiratory diseases^26,27^, although these studies lacked a proper systemic approach and statistical support. In the present study, we assessed the evidence of an association between the presence of the CanineCV genome and dogs with respiratory symptoms. The results demonstrated a significant correlation between CanineCV-positive dogs and the presence of respiratory symptoms (*p* = 0.013). This finding supports our hypothesis that CanineCV could be a pathogen associated with canine respiratory disease. The odds ratio of 5.606 between CanineCV positivity in the respiratory and healthy groups also suggests that dogs showing respiratory signs are 5.6 times more prone to being positive for CanineCV in comparison with healthy dogs.

CanineCV occurrence in dogs with respiratory disease in this epidemiological study was 13.16% (15/114), which is lower than in a previous report (30%, 6/20)^27^. This difference might be the result of the different location used for sample collection, the larger sample size, and the age variation of dogs with respiratory disease in this study.

In the present study, the CanineCV detection rate was significantly higher in junior dogs than older dogs (*p* = 0.007). The odds ratio was 3.83, indicating that the junior dogs were approximately 3.8 times more likely to be positive for CanineCV compared with the adult and senior dogs. This finding is in concordance with several reports^18,22^. Among junior dogs, it was noticeable that the number that were CanineCV-positive was higher at ≤ 6 months of age (Supplementary Table S1).

In the CanineCV-positive group, 52.94% (9/17) of dogs were co-detected with CIRDC viruses; however, a confounding effect of these viruses was not found (*p* = 1.00) indicating that CanineCV has its own potential role in the development of canine respiratory symptoms. The observation of coinfection has been reported in several studies, and it was believed that *Circovirus* infection was prone to coinfection with other pathogens^21–23,32^, resulting in impairment of immune response^28,29,31^. Whether CanineCV plays a role in the induction of the immunosuppressive condition or in the augmentation of target inflammatory cells when coinfected with other viral infections requires further investigation. Apart from CIRDC viruses, several respiratory bacteria have been linked as causes of canine respiratory disease; additional bacterial culture might be conducted to clarify the role of single CanineCV infection.

Identifying CanineCV genomic materials from oropharyngeal swabs might be affected by fecal–oral contamination; however, the virus could also be detected from nasal swabs. This finding implies that CanineCV can be examined from respiratory secretions in dogs suffering from respiratory illness. To support evidence of CanineCV in association with respiratory diseases, we found the presence of CanineCV tropism in inflammatory cells localized in lung parenchyma and alveolar areas, where it presented in inflammation of the CIRDC-negative dog with a history of respiratory disease. The CanineCV DNA was also presented within lymphoid cells in lymphoid follicles of respiratory-related lymphoid organs. Similar features were demonstrated in previous studies in which ISH-positive cells were lymphoid cells or macrophages^24,27^. Demonstration of CanineCV nucleic acid within inflammatory mononuclear cells in lesion areas in pulmonary parenchyma leads to speculation that (i) the infected mononuclear cells could have reached the lungs due to the bacterial infection and local inflammation, (ii) the virus might contribute either direct or indirect effects in canine respiratory disease by inducing immunocompromise leading to secondary infection, or (iii) the virus might play a role in the systemic inflammatory response^33^ that leads to an upregulation of pulmonary alveolar macrophages^34^ found in this study or an overwhelming number of cytokine bombs, which were found in the PCV-2 infection^35^. In addition, the CanineCV DNA labeling was found in alveolar lining epithelia and capillary endothelial lining, where necrotic alveolitis was presented. These pathological features are similar to descriptions of the PCV-2 infection in pigs^36,37^. Detection of the CanineCV genome in various tissues by qPCR indicates the CanineCV tissue distribution. A higher tissue viral load in tracheobronchial lymph nodes (dog nos. 2 and 4) together with ISH-positive signals in the tracheobronchial lymph nodes could reflect the tissue preference of the virus. Thus, diffuse infection in regional lymph nodes of respiratory system might disperse and cause respiratory disease, as presented in this study. Because the dog died as a possible consequence of bacterial bronchopneumonia that could have led to the spreading of the virus in the lungs, further investigation of more CanineCV-positive lung sections and identification of CanineCV cellular tropism will facilitate this implementation.

Phylogenetic analysis based on whole genome sequencing provided evidence that CanineCV was divided into five genotypes, consistent with previous reports^38,39^. All CanineCV strains obtained from this study were clustered within the CanineCV-4 genotype, along with the earlier detected Thai strains, suggesting that the CanineCV-4 genotype was currently circulating among the Thai dog population. Even though the DNA virus has a lower mutation rate compared with the RNA virus, CanineCV has undergone genetic recombination for its evolution, as indicated by several reports, including a first report of recombinant CanineCV sequences were detected from infected dogs in Thailand^23,27,40^. In addition, we found that some of our CanineCV strains were separated into different phylogenetic clades based on the *Rep* and *Cap* genes. Thus, we further investigated with recombination analysis. CanineCV recombination was found where the recombination breakpoint was presented in the *Rep* gene. This finding is in accordance with previous findings^27^. Genetic recombination occurs when the host is coinfected with two or more virus strains, and recombination events can be found within the gene or intergenic region^23,40^. Furthermore, the recombination breakpoint analysis of PCV2 demonstrated that it occurred to a lesser extent in the *Cap* gene than in the *Rep* gene because of the viral assembly protein disruption^41^.

In conclusion, we reported the first study of CanineCV-associated respiratory disease in domestic dogs. The virus can be detected from nasal and oropharyngeal swabs of dogs, together with evidence of viral tropism in cells circulating in lung tissue that correspond to pathological lesions, implying a possible transmission pathway of the agent and potential contributory role of CanineCV in the canine infectious respiratory disease, which requires further elucidation. Viral viability and infectivity testing are required to confirm the postulation. Further research is necessary to elucidate the role of CanineCV pathogenesis in respiratory diseases in dogs. In addition, we found that the CanineCV-4 genotype is distributed among the Thai dog population, and that one genotype has undergone genetic recombination for its adaptation. Intensive molecular monitoring and surveillance of CanineCV mutations are necessary for disease control and prevention.

## Materials and methods

### Animal and ethics

A total of 190 dogs, comprising both healthy dogs (*n* = 76) and dogs with respiratory symptoms (*n* = 114), were included in this study. The research assessed nasal swab (NS) and oropharyngeal swab (OS) collected between January and November 2020. The samples from the healthy group were collected from clinically healthy dogs during routine dental scaling at the Veterinary Teaching Hospital, Faculty of Veterinary Medicine, Kasetsart University, Bangkok, Thailand. Samples from dogs with respiratory signs were collected from owned dogs that visited the Small Animal Teaching Hospital, Faculty of Veterinary Science, Chulalongkorn University, Bangkok and vicinities, Thailand. Clinical signs of dogs included sneezing, coughing, nasal discharge, and bronchopneumonia which was diagnosed based on thoracic radiography. Dogs showing respiratory signs caused by a nasal foreign body, heart disease, or functional tracheal disease; dogs exhibiting enteric diseases; and dogs that received a vaccination within the 4 weeks prior to sampling were excluded.

Essential signalments including sex, breed, sterilization status, and respiratory clinical signs were recorded for further analysis. Dogs’ age was classified as followed: junior dogs (age < 1.5 years), adult dogs (age ≥ 1.5 to < 6 years), and senior dogs (age ≥ 6 years)^42,43^. To support the possible potential role of CanineCV in respiratory disease, dogs that had died from bronchopneumonia submitting for routine necropsy at Department of Pathology, Faculty of Veterinary Science, Chulalongkorn University, were included in the study. This research was approved by the Institutional Animal Care and Use Committee (IACUC) (No. 2031014) and the Institutional Biosafety Committee (IBC) (No. 1931036) of Chulalongkorn University. This study is reported in accordance with ARRIVE guidelines.

### Sample collection and DNA extraction

For the NS sampling site, the swabs (Puritan, Guilford, USA) were inserted into the nostril to about 3 cm or shallower according to the dog breed, whereas OSs were placed on the oropharynx around the tonsils and then rolled gently. NSs and OSs collected from all dogs were immersed in 1% (v/v) sterile phosphate buffer saline (PBS) and then subjected to genomic extraction immediately using the Viral DNA/RNA extraction kit (Geneaid, Ltd., Taipei, Taiwan) following the manufacturer’s protocol. The extracted nucleic acids were quantified and qualified on the basis of a 260/280 absorbance ratio using a Nanodrop® Lite spectrophotometer (Thermo Fisher Scientific Inc., Waltham, MA, USA). The extracted samples were kept at − 80 °C until used.

A total of 15 lungs from necropsied dogs suffering from bronchopneumonia were obtained and divided for molecular assays and pathological examination. For molecular detection, the frozen lung tissues were minced, and homogenized in 1% PBS by a tissue homogenizer and clarified by centrifugation at 10,000×*g* for 3 min. The supernatants were collected for genomic extraction as mentioned earlier and stored at − 80 °C until assayed. The remaining part of the lungs was fixed in 10% neutral buffered formalin for pathological purposes. Lung tissues from necropsied dogs that died from non-respiratory disease were included for CanineCV detection by cPCR, comprising dogs suffering from cardiovascular failure (n = 13) and accidentally traumatic injury (n = 15).

### Virus-associated Canine Infectious Respiratory Disease Complex (CIRDC) detection

Common canine respiratory viruses, including canine influenza virus (CIV), canine parainfluenza virus (CPIV), canine distemper virus (CDV), canine respiratory coronavirus (CRCoV), canine adenovirus type 1 and 2 (CAdV-1 & 2), and canine herpesvirus 1 (CaHV-1), were screened in the samples (NS and OS) from both healthy and respiratory dogs, as well as homogenized lung tissues, using multiplex reverse-transcription (RT)-PCR/PCR assays as previously described^44^. The information regarding the virus-associated CIRDC tests was used for further interpretation.

### CanineCV detection and full-length genome amplification

The nucleic acids extracted from both studied groups were tested for CanineCV genome by conventional PCR (cPCR) using Gotaq Green Master mix (Promega, WI, USA) and specific primers (F605-R1041 and F1022-R1538), to amplify the partial *Rep* and *Cap* genes (Table 4). The thermocycling condition consisted of an initial denaturation at 95 °C for 2 min, followed by 35 cycles at 95 °C for 30 s, 55 °C for 30 s, and 72 °C for 30 s, and then a final extension at 72 °C for 7 min. To complete the full-length genome of CanineCV, multiple primer pairs (F1349 or F1372-R110 and F2014-R764) were used to amplify the CanineCV sequences (Table 4). The thermocycling condition was set as the same condition described above, except for the annealing temperature, which was changed to 58 °C for 30 s with an extension time at 72 °C for 45 s. The positive control for CanineCV cPCR was retrieved from a previous publication^27^. A no-template control (NTC) was used as the negative control. The PCR products were separated on 1.5% (w/v) agarose gel containing ethidium bromide and were visualized under a UV transilluminator.

**Table 4:** Primers used for the PCR-based canine circovirus (CanineCV) detection and sequencing.

Primer name	Primer sequence (5’–3’)	Product size (bp)	NCBI reference	References
CanineCV-F605	AATGGTGGGAYGGYTACGATGG	437	MG737378	Piewbang et al.^27^
CanineCV-R1041	AAGGGGGGTGAACAGGTAAAC			Piewbang et al.^27^
CanineCV-F1022	TTTACCTGTTCACCCCCCTTCGA	517	MG737378	Piewbang et al.^27^
CanineCV-R1538	GGAAGAGGYAATGCTACAAGATCA			Piewbang et al.^27^
CanineCV-F1349	TGTCCTGAGTGAACATTGGTTTTGG	845	MZ826145	This study
CanineCV-F1372	GGTGTCAGWCCCCTTTTGAATCCAG	822	MZ826142	This study
CanineCV-R110	TCCGGCGCRAGGTTCTTCA		MT740194	Tuong et al.^39^
CanineCV-F2014	GTATTACCCGGCACCTCGTC	814	MT740194	Tuong et al.^39^
CanineCV-R764	GTGATGAATACACGGCGGGC		MZ826142	This study
CanineCV-F247	CGCGGCCATTTTGAGCCTGCTC	254	ON863358	This study
CanineCV_R502	TCCGGGGCGTGGTCATCTCC		ON863358	This study

To confirm the CanineCV nucleotide sequences, the PCR products were purified using NucleoSpin Extract II (Macherey–Nagel, Düren, Germany) and submitted for bidirectional Sanger sequencing (Macrogen Inc., Seoul, South Korea). The obtained sequences were initially analyzed by alignment with previously published CanineCV genomes available in the GenBank database using BLASTn analysis. For full-length genome characterization, several genome products from multiple cPCRs with specific primer pairs were visualized, purified, and submitted for Sanger sequencing as described above. The derived genetic sequences were initially blasted with the previous CanineCV sequences deposited in GenBank to confirm the presence of CanineCV genomes. Subsequently, the derived genetic sequences were aligned and assembled using BioEdit software package version 7.2 with the ClustalW function.

### Presence of CanineCV in tissues by qPCR

To quantify the viral load in different tissues (Supplementary Table S6) of CanineCV positive carcasses, SYBR Green-based qPCR was performed using the KAPA SYBR® Fast qPCR Master Mix (2X) Universal kit (KAPABIOSYSTEMS, Sigma-Aldrich, Cape Town, South Africa). The reaction consisted of a 250 nM final concentration of each primer (F247–R502) (Table 4), which were used to amplify the partial *Rep* gene, giving a product size of 254 bp. The amplification was processed in a Rotor-Gene Q real-time PCR cycler (Qiagen GmbH, Hilden, Germany) with the protocol of initial denaturation at 95 °C for 3 min followed by 40 cycles of 95 °C for 10 s and 60 °C for 30 s to acquire the fluorescence A green. Then, melt curve analysis was performed to define the melting temperature of the amplicon. A positive result was expected to melt at 85.0 °C to 85.5 °C when the temperature was increased from 60 to 95 °C. The software reported the cycling A green and melt A green compared with the NTC and positive control retrieved from the positive clinical samples confirmed by sequencing. An output Ct value was used to estimate the viral copy number in each organ.

### Phylogenetic and recombination analyses

The phylogenetic tree was constructed using MEGA software package version 7.0. The maximum likelihood (ML) method, based on the general time reversible model (GTR) with a gamma distribution and invariable sites (G + I) together with 1,000 bootstrap replicates, was used to evaluate the relationship between these obtained CanineCV strains and the other strains deposited in the GenBank database. Additionally, the CanineCV alignment was subjected to recombination analysis using the Recombination Detection Program software package version 4.0 (RDP4). The RDP included seven recombination detection methods (RDP, GeneConv, BootScan, MaxChi, Chimera, SiScan, and 3Seq) to analyze the recombination breakpoint. A potential recombination sequence was accepted with a *p*-value of ≤ 10^–10^ from at least four out of the seven methods. Subsequently, the potential recombination strains derived from the RDP analysis were subjected to similarity plot and Bootscan analysis using SIMPLOT software package v. Beta 4.94 to additionally confirm the presence of the recombination breakpoint. The program was set and analyzed according to previous publications^27^.

### Pathological examination

After fixing the fresh lung tissues in 10% neutral buffered formalin for 48 h, tissues were further histologically processed and embedded in paraffin wax, sectioned at a 4 µm-thickness, and stained with Hematoxylin and Eosin (HE). Microscopic findings were inspected by Thai board-certified veterinary pathologist (ST).

### In situ hybridization (ISH)

The CanineCV-PCR positive lungs were further investigated for the CanineCV genome in the tissues by ISH. The digoxigenin (DIG)-labeled probe covering 437 bp of the partial CanineCV *Rep* gene was constructed by utilizing the PCR DIG probe synthesis kit (Roche Diagnostics, Basel, Switzerland) according to the manufacturer’s protocol. Briefly, the CanineCV-DIG probe was synthesized with the same thermal cycling conditions described in cPCR for the partial *Rep* gene. The DIG-labeled oligonucleotides were used instead of the normal oligonucleotides. Then, the constructed CanineCV-DIG probe was observed by visualizing the product size on 1.5% (w/v) agarose gel electrophoresis.

After sections were deparaffinized and rehydrated, they were then pretreated with 0.2 N hydrochloric acid (HCl) at room temperature for 20 min, followed by citrate buffer (pH 6) at 95 °C for 20 min. The slides were incubated with 10 ng/mL proteinase K (VWR, Radnor, PA, USA) in 1X Tris-NaCl-EDTA (TNE) buffer at 37 °C for 20 min and soaked with 0.4% cold formaldehyde solution. Then, slides were prehybridized with prehybridization buffer containing 50% (v/v) formamide in 4X saline-sodium citrate (SSC) buffer at 37 °C for at least 15 min and subsequently incubated with a hybridization buffer containing 20X SSC, 5X Denhardt’s solution, 100 ug/mL salmon sperm DNA, 0.5% (w/v) sodium dodecyl sulfate, and 10 ng of CanineCV-DIG-labeled probe per slide at 50 °C overnight in the slide incubator. After incubation, the slides were stringently washed in a series of 2X SSC at 37 °C, 1X SSC at 42 °C, and 0.5X SSC at 42 °C, each for 15 min, respectively. After that, the slides were then incubated in 5% bovine serum albumin (BSA) for non-specific binding blocking at room temperature for 30 min. The hybridization reactions were visualized by incubating with 1:200 anti-DIG-AP Fab fragments (Roche, Mannheim, Germany) in 1X blocking solution, and the chromogenic signals were developed using PermaRed/AP (Diagnostic BioSystems, CA, USA) applied in a dark chamber at room temperature for 40 min. Slides were then counterstained with hematoxylin. The dark red dots presented within the cellular structure were considered as positive results, whereas the CanineCV PCR-negative section and unrelated probe, canine bocavirus (CBoV) probe^45^, were used as negative controls.

### Statistical analysis

The associations between the presence of CanineCV and other variable data, including age, sex, breed, sterilization status, sampling route, co-detection with CIRDC viruses, and clinical symptoms of sampled dogs, were calculated using Pearson’s chi-squared test or Fisher’s exact test depending on the population size being assessed in each factor. The output data were considered statistically significant at a *p*-value < 0.05. The odds ratio was then calculated to measure the strength of association between each factor and CanineCV occurrence. The statistical analyses were conducted using SPSS version 22.0 (SPSS Inc., Chicago, IL, USA).

### Ethics statement

The authors confirm that the ethical policies of the journal, as noted on the journal’s author guidelines page, have been adhered to and the appropriate ethical review committee approval has been received by the Chulalongkorn University Animal Care and Use Committee (No. 2031014). All procedures were done in accordance with the relevant guidelines and regulations. Authors confirm that this study is reported in accordance with ARRIVE guidelines.

## Supplementary Information

Supplementary Information.

## Data Availability

All the data supporting our findings is contained within the manuscript. Seven full-length coding CanineCV sequences have been deposited in NCBI GenBank under accession MZ826142-MZ826148.
