# Supplementary material for: Epidemiology, genetic diversity, and association of canine circovirus infection in dogs with respiratory disease
Source: Sci Rep. 2022 Sep 14;12:15445. doi: 10.1038/s41598-022-19815-z (PMC9472715; doi:10.1038/s41598-022-19815-z)
Supplement: Supplementary file 1 — Supplementary Information. [file 41598_2022_19815_MOESM1_ESM.docx]

**Epidemiology, genetic diversity, and association of canine circovirus infection in dogs with respiratory disease**

**Wichan Dankaona^1,2^, Emmita Mongkholdej^3^, Chakkarin Satthathum^3^,** **Chutchai Piewbang^1,2^, Somporn Techangamsuwan^1,2,*^**

^1^ Department of Pathology, Faculty of Veterinary Science, Chulalongkorn University, Bangkok 10330, Thailand

^2^ Animal Virome and Diagnostic Development Research Group, Faculty of Veterinary Science, Chulalongkorn University, Bangkok 10330, Thailand

^3^ Division of Dentistry and Maxillofacial Surgery, Kasetsart University Veterinary Teaching

Hospital, Bangkok 10900, Thailand

**Supplementary Table S1** Demographic data of Canine circovirus positive dogs in healthy (H), respiratory (R), and necropsied dogs

| **Dogs** | **Age** | **Sex** | **Breed** | **Sterilization** | **Clinical signs^†^** | **Status^‡^** | **Detected sample^§^** | **Co-detected virus**^¶^ |
| --- | --- | --- | --- | --- | --- | --- | --- | --- |
| H026 | 1 year 6 months | Female | Yorkshire Terrier | Intact | NR | A | NS, OS | - |
| H070 | 5 years | Female | Maltese | N/A | NR | A | NS, OS | CRCoV |
| R006 | 4 months | Female | Siberian Husky | Intact | S, ND, C | N/A | NS | - |
| R009 | 5 months | Female | Maltese | Intact | C | A | NS, OS | CaHV-1 |
| R010 | 5 months | Male | Maltese | Intact | S, ND, C | A | NS, OS | - |
| R018 | 12 years | Female | Yorkshire Terrier | Intact | S, ND | D | NS | - |
| R025 | 7 months | Female | Welsh Corgi | Intact | S, ND, C, B | A | NS, OS | CPIV |
| R036 | 5 months | Female | Yorkshire Terrier | Intact | S, ND, C | D | NS, OS | CAdV-2 |
| R037 | 5 months | Female | Shih Tzu | Intact | S, ND, C | A | NS, OS | - |
| R051 | 3 months | Female | Mixed | Neutered | ND, C | N/A | NS, OS | CDV |
| R067 | 2 months | Male | Mixed | Intact | ND, C | N/A | OS | - |
| R076 | 2 years | N/A | Mixed | Neutered | ND, C | N/A | OS | - |
| R077 | 1 year 6 months | Female | Mixed | Neutered | ND, C | N/A | NS | CRCoV |
| R081 | 1 year 8 months | Female | Mixed | Neutered | ND | N/A | NS, OS | CDV |
| R099 | 2 years 6 months | Male | Mixed | Neutered | ND | N/A | NS | CaHV-1 |
| R101 | 1 year | Male | Mixed | Neutered | ND | N/A | OS | - |
| R103 | 6 months | Female | Mixed | Intact | S | N/A | NS, OS | CaHV-1, CRCoV |
| Dog No. 1 | 10 days | Male | Mixed | Intact | B | D | N/A | - |
| Dog No. 2 | 2 years | Male | Mixed | Intact | B | D | N/A | - |
| Dog No. 3 | 4 months | Male | Chow Chow | Intact | C, B | D | N/A | - |
| Dog No. 4 | 8 months | Female | Pomeranian | Intact | B | D | N/A | - |

^†^No respiratory sign (NR), sneezing (S), nasal discharge (ND), coughing (C), bronchopneumonia (B)

^‡^Alive (A), Death (D), No data available (N/A)

^§^Nasal swab (NS), Oropharyngeal swab (OS)

^¶^Canine herpesvirus 1 (CaHV-1), canine distemper virus (CDV), canine respiratory coronavirus (CRCoV), canine parainfluenza virus (CPIV), and canine adenovirus type 2 (CAdV-2)

**Supplementary Table S2** Other respiratory viruses^1^ detected by multiplex RT-PCR/PCR assays in canine circovirus-negative dogs with respiratory illness from swab samples

| **Single infection** | **(n=45)** |
| --- | --- |
| CaHV-1  CRCoV  CDV  CPIV  CAdV-2 | 17  14  9  3  2 |
| **Dual infection** | **(n=10)** |
| CDV + CaHV-1  CDV + CAdV-2  CRCoV + CAdV-2  CRCoV + CaHV-1  CAdV-2 + CaHV-1 | 6  1  1  1  1 |
| **Triple infection** | **(n=1)** |
| CDV + CRCoV + CAdV-2 | 1 |
| **Total** | 56 |

^†^Canine herpesvirus 1 (CaHV-1), canine distemper virus (CDV), canine respiratory coronavirus (CRCoV), canine parainfluenza virus (CPIV), and canine adenovirus type 2 (CAdV-2)

**Supplementary Table S3** Factors associated with canine circovirus (CanineCV)-positive dogs

| **Factor** | **Category** | **Number examined** | **Number positive (%)** | **Statistical test** | ***p* value** |
| --- | --- | --- | --- | --- | --- |
| Age | Junior (< 1.5 years)  Adult (1.5 - 6 years)  Senior (≥ 6 years) | 57  57  76 | 10 (17.54%)  6 (10.53%)  1 (1.32%) | Pearson Chi-Square | 0.005 |
|  | Junior (<1.5 years)  Older (≥ 1.5 years) | 57  133 | 10 (17.54%)  7 (5.26%) |  | 0.007 |
| Sex | Male  Female | 72  114 | 4 (5.56%)  12 (10.53%) |  | 0.239 |
| Breed | Pure breed  Mixed breed | 113  77 | 9 (7.97%)  8 (10.39) |  | 0.605 |
| Sterilization status | Intact  Neutered | 79  100 | 10 (12.66%)  6 (6.00%) |  | 0.121 |
| Disease status | Respiratory  Healthy | 76  114 | 15 (13.16%)  2 (2.63%) |  | 0.013 |
| Sampling route | Nasal swab  Oral swab | 17  17 | 14 (82.35)  13 (76.47%) | Fisher’s Exact test | 0.541 |
| Coinfection | Present  Absent | 17  17 | 9 (52.94%)  8 (47.06%) |  | 1.000 |

**Supplementary Table S4a** Nucleotide sequence identity of seven full-length genomes of Canine circovirus (CanineCV) from this study

| Sequences | CanineCV_R009 | CanineCV_R010 | CanineCV_R025 | CanineCV_H026 | CanineCV_H070 | CanineCV_R081 | CanineCV_R103 |
| --- | --- | --- | --- | --- | --- | --- | --- |
| CanineCV_R009 |  |  |  |  |  |  |  |
| CanineCV_R010 | 0.999 |  |  |  |  |  |  |
| CanineCV_R025 | 1 | 0.999 |  |  |  |  |  |
| CanineCV_H026 | 0.963 | 0.962 | 0.963 |  |  |  |  |
| CanineCV_H070 | 0.953 | 0.953 | 0.953 | 0.952 |  |  |  |
| CanineCV_R081 | 0.963 | 0.962 | 0.963 | 0.954 | 0.955 |  |  |
| CanineCV_R103 | 0.962 | 0.962 | 0.962 | 0.955 | 0.955 | 0.999 |  |

**Supplementary Table S4b** Nucleotide sequence identity (lower half) and amino acid identity (upper half) of seven *Replicase* genes of Canine circovirus (CanineCV) from this study

| Sequences | CanineCV_R009 | CanineCV_R010 | CanineCV_R025 | CanineCV_H026 | CanineCV_H070 | CanineCV_R081 | CanineCV_R103 |
| --- | --- | --- | --- | --- | --- | --- | --- |
| CanineCV_R009 |  | 1 | 1 | 0.963 | 0.957 | 0.966 | 0.966 |
| CanineCV_R010 | 1 |  | 1 | 0.963 | 0.957 | 0.966 | 0.966 |
| CanineCV_R025 | 1 | 1 |  | 0.963 | 0.957 | 0.966 | 0.966 |
| CanineCV_H026 | 0.959 | 0.959 | 0.959 |  | 0.966 | 0.96 | 0.96 |
| CanineCV_H070 | 0.952 | 0.952 | 0.952 | 0.953 |  | 0.976 | 0.976 |
| CanineCV_R081 | 0.956 | 0.956 | 0.956 | 0.95 | 0.958 |  | 1 |
| CanineCV_R103 | 0.956 | 0.956 | 0.956 | 0.95 | 0.958 | 1 |  |

**Supplementary Table S4c** Nucleotide sequence identity (lower half) and amino acid identity (upper half) of seven *Capsid* genes of Canine circovirus (CanineCV) from this study

| Sequences | CanineCV_R009 | CanineCV_R010 | CanineCV_R025 | CanineCV_H026 | CanineCV_H070 | CanineCV_R081 | CanineCV_R103 |
| --- | --- | --- | --- | --- | --- | --- | --- |
| CanineCV_R009 |  | 1 | 1 | 0.974 | 0.955 | 0.97 | 0.97 |
| CanineCV_R010 | 0.998 |  | 1 | 0.974 | 0.955 | 0.97 | 0.97 |
| CanineCV_R025 | 1 | 0.998 |  | 0.974 | 0.955 | 0.97 | 0.97 |
| CanineCV_H026 | 0.958 | 0.956 | 0.958 |  | 0.94 | 0.944 | 0.944 |
| CanineCV_H070 | 0.943 | 0.943 | 0.943 | 0.936 |  | 0.944 | 0.944 |
| CanineCV_R081 | 0.959 | 0.959 | 0.959 | 0.943 | 0.938 |  | 1 |
| CanineCV_R103 | 0.958 | 0.958 | 0.958 | 0.944 | 0.937 | 0.998 |  |

**Supplementary Table** **S5** Support of recombination event detected by the different methods implemented by Recombination Detection Program (RDP)

| **Event**  **No.** | **Strains** | **Parents** | | **Breakpoint (Nt)** | | **Average *p*-value** | | | | | | |
| --- | --- | --- | --- | --- | --- | --- | --- | --- | --- | --- | --- | --- |
|  |  | Major | Minor | Start | End | RDP | GeneConv | BootScan | MaxChi | Chimera | SiScan | 3Seq |
| 1 | CB6293/2-14 | 390 | CB6293/1-14 | 2055 | 1084 | 1.55E^-10^ | 1.67E^-28^ | 2.10E^-18^ | 1.51E^-19^ | 4.34E^-19^ | 4.44E^-22^ | 1.23E^-41^ |
| 2 | WM46 | K1 | WM83 | 978 | 2000 | 2.79E^-32^ | 9.27E^-24^ | 1.14E^-27^ | 5.84E^-20^ | - | 3.97E^-33^ | 9.12E^-12^ |
| 3 | WM83 | GL51 | WM74 | 941 | 2027 | 3.73E^-29^ | 2.45E^-21^ | 3.33E^-04^ | 1.92E^-20^ | - | 2.14E^-34^ | 9.12E^-12^ |
| 4 | C85 | C79 | GL33 | 1750 | 100 | 3.52E^-20^ | 2.17E^-17^ | 6.55E^-15^ | 5.93E^-12^ | 3.80E^-11^ | 5.84E^-08^ | 9.12E^-12^ |
| 5 | TE6685/2-13 | TE7482-13 | NM_N73/2019 | 2030 | 249 | 6.23E^-18^ | 9.14E^-10^ | 2.16E^-14^ | 1.37E^-07^ | 1.19E^-08^ | - | 1.85E^-19^ |
| 6 | XF16 | C85 | PE8575/2-13 | 636 | 894 | 6.45E^-18^ | 1.53E^-15^ | 4.48E^-13^ | 5.30E^-09^ | 1.30E^-08^ | 5.89E^-11^ | 9.21E^-19^ |
| 7 | AH-1/2019 | CanineCV_R010 | GL33 | 1740 | 404 | 7.20E^-17^ | 8.14E^-05^ | 2.16E^-11^ | 5.19E^-14^ | 8.23E^-15^ | 1.57E^-12^ | 1.79E^-03^ |
| 8 | CanineCV_H070 | UCD3-478 | 182 | 354 | 1071 | 1.17E^-16^ | 8.99E^-15^ | 1.89E^-10^ | 6.33E^-13^ | 4.67E^-14^ | 7.15E^-15^ | 8.38E^-18^ |
| 9 | WM66 | WM79 | WM74 | 2020 | 795 | - | 2.90E^-14^ | 2.20E^-13^ | 3.56E^-10^ | 4.70E^-10^ | 8.80E^-13^ | 5.61E^-26^ |
| 10 | AH-1/2019 | UCD3-478 | LA280 | 405 | 1186 | 5.70E^-08^ | 2.51E^-05^ | 1.94E^-07^ | 2.70E^-11^ | 5.76E^-12^ | 8.79E^-14^ | 7.63E^-17^ |

- = No recombination detection

**Supplementary Table S6** Viral load in Ct values and pathological findings of canine circovirus (CanineCV)-positive necropsied dogs

| **Animals** | **Samples** | **Ct value** | **Pathological findings** |
| --- | --- | --- | --- |
| Dog No. 1 | Tracheobronchial lymph node | 27.51 | Mild hemorrhagic histiocytic lymphadenitis |
|  | Lung | 25.36 | Multifocal hemorrhagic histiocytic pneumonia |
|  | Liver | 10.17 | Severe generalized perivasculitis |
|  | Spleen | 24.34 | Splenic lymphoid depletion and necrosis |
|  | Kidney | negative | Diffuse membranous glomerulonephritis |
|  | Intestine | 28.78 | No remarkable lesion |
| Dog No. 2 | Tracheobronchial lymph node | 15.94 | Moderate hemorrhagic histiocytic lymphadenitis |
|  | Trachea | 22.37 | No remarkable lesion |
|  | Bronchus | negative | Mild chronic bronchitis |
|  | Lung | 24.02 | Hemorrhagic pyogranulomatous pneumonia |
|  | Liver | 29.36 | Hepatic glycogen degeneration and congestion |
|  | Spleen | 27.02 | Splenic extramedullary hematopoiesis |
|  | Kidney | negative | Mild chronic interstitial nephritis |
|  | Intestine | 27.37 | No remarkable lesion |
|  | Mesenteric lymph node | 20.62 | Mild lymphoid depletion |
| Dog No. 3 | Tracheobronchial lymph node | 25.75 | Moderate hemorrhagic histiocytic lymphadenitis |
|  | Trachea | negative | No remarkable lesion |
|  | Bronchus | negative | No remarkable lesion |
|  | Lung | 24.18 | Hemorrhagic pyogranulomatous pneumonia |
|  | Liver | 24.68 | Liver cells atrophy |
|  | Spleen | negative | Hemosiderosis |
|  | Kidney | negative | No remarkable lesion |
|  | Intestine | 27.49 | No remarkable lesion |
|  | Pancreatic lymph node | 24.24 | No remarkable lesion |
|  | Submandibular lymph node | 27.26 | Mild lymphoid depletion |
| Dog No. 4 | Tracheobronchial lymph node | 22.19 | Severe hemorrhagic histiocytic lymphadenitis |
|  | Trachea | 22.51 | Suppurative tracheitis |
|  | Bronchus | 23.46 | Severe suppurative necrotizing bronchitis |
|  | Lung | 24.24 | Severe diffuse suppurative hemorrhagic pneumonia |
|  | Liver | 23.78 | Hepatic congestion |
|  | Spleen | 29.83 | Splenic extramedullary hematopoiesis |
|  | Kidney | 28.76 | Renal congestion |
|  | Intestine | 29.75 | No remarkable lesion |
|  | Mesenteric lymph node | 20.29 | Mild lymphoid depletion |


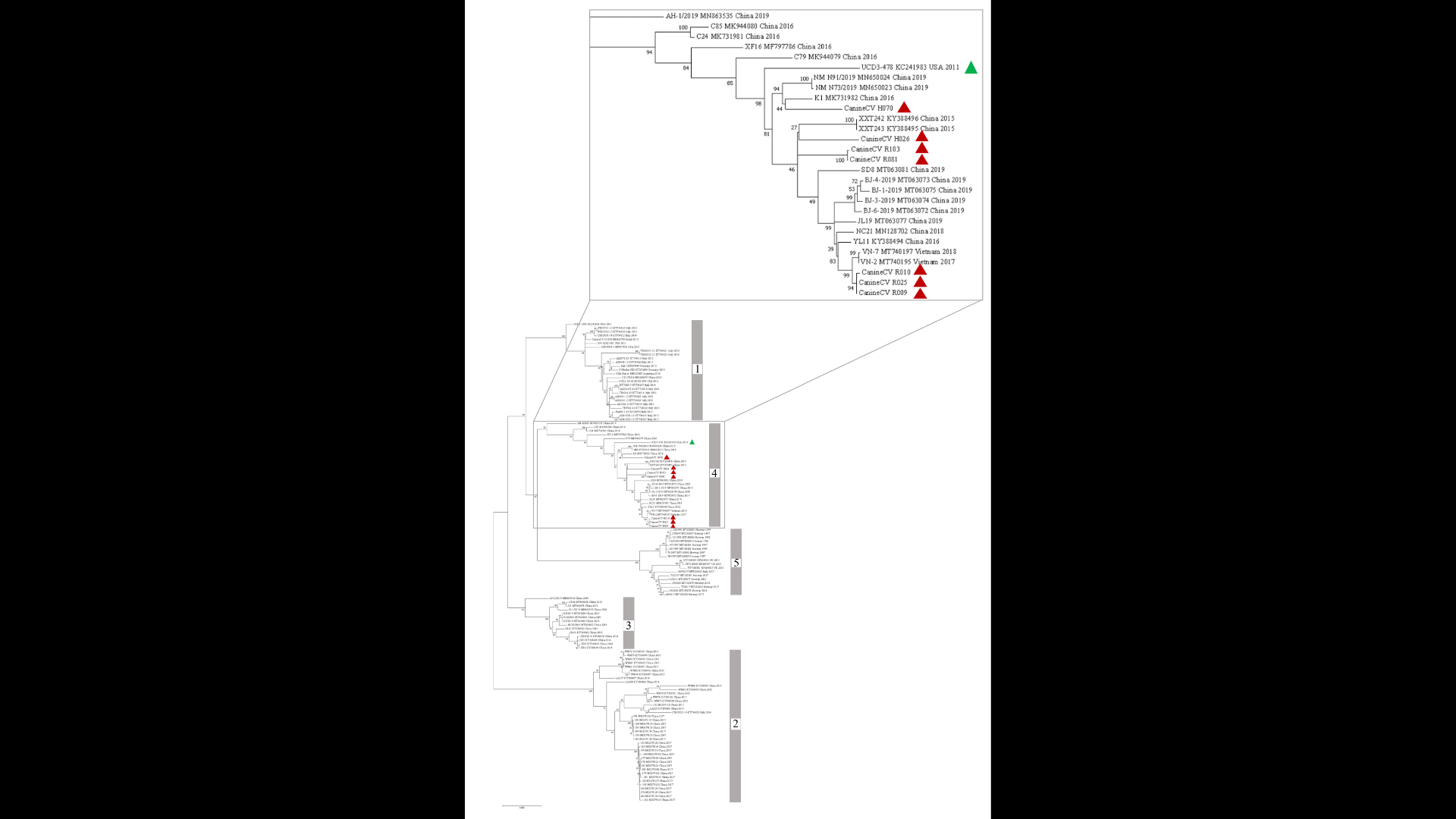


**Supplementary Figure S1.** Maximum likelihood phylogenetic tree reconstructed using the combined nucleotide sequences at position 1-353 and 1072-2063 of canine circovirus (CanineCV). The reliability of the phylogeny was evaluated using the 1,000 bootstrap test replications ( represents the CanineCV strains from this study and represents the major parent of CanineCV_H070).

**
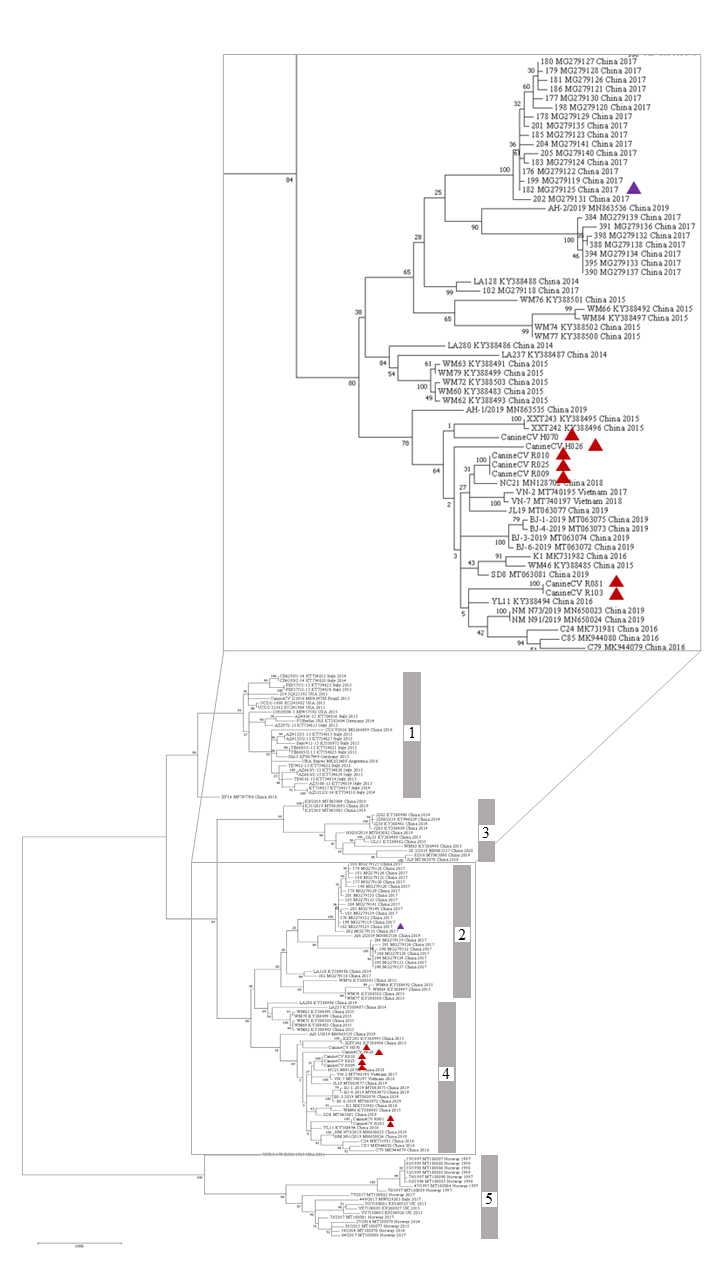
**

**Supplementary Figure S2**. Maximum likelihood phylogenetic tree reconstructed using the nucleotide sequences at position 354-1071 of canine circovirus (CanineCV). The reliability of the phylogeny was evaluated using the 1,000 bootstrap test replications ( represents the CanineCV strains from this study and represents the minor parent of CanineCV_H070).

**
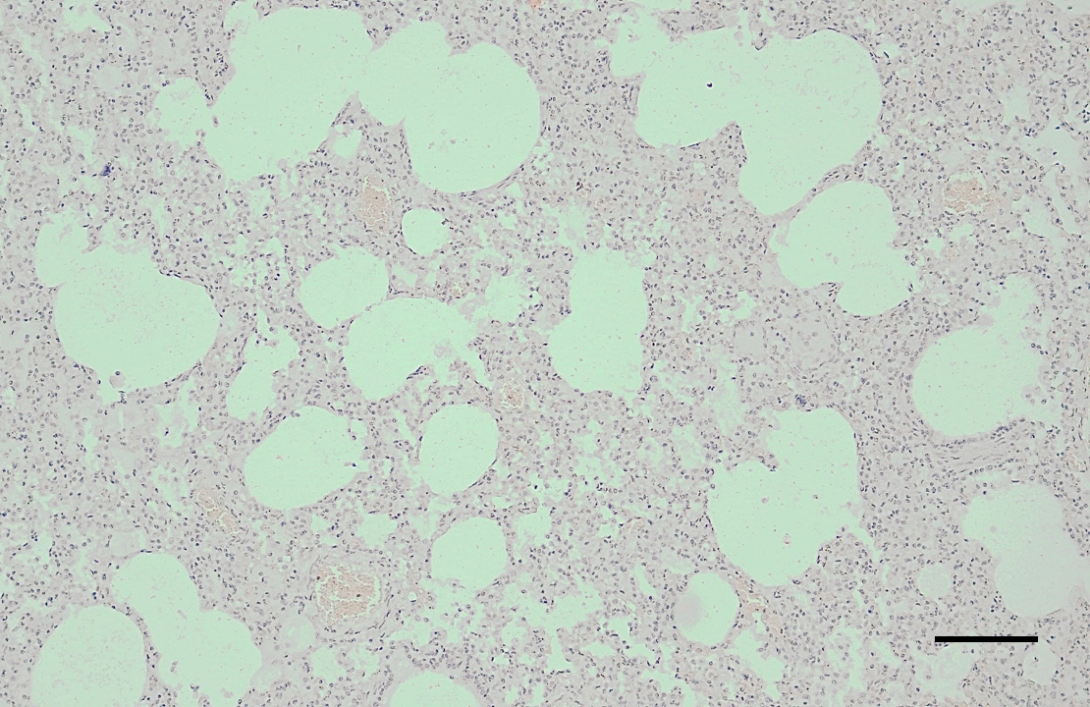
**

**Supplementary Figure S3.** Negative control for in situ hybridization. Canine circovirus (CanineCV) DNA labeling was not observed in CanineCV PCR-negative section. Bar: 250 µm.
